# Insect-Inspired Navigation Algorithm for an Aerial Agent Using Satellite Imagery

**DOI:** 10.1371/journal.pone.0122077

**Published:** 2015-04-15

**Authors:** Douglas D. Gaffin, Alexander Dewar, Paul Graham, Andrew Philippides

**Affiliations:** 1 Department of Biology, University of Oklahoma, Norman, Oklahoma, United States of America; 2 School of Life Sciences, University of Sussex, Brighton, United Kingdom; 3 Department of Informatics, University of Sussex, Brighton, United Kingdom; Lund University, SWEDEN

## Abstract

Humans have long marveled at the ability of animals to navigate swiftly, accurately, and across long distances. Many mechanisms have been proposed for how animals acquire, store, and retrace learned routes, yet many of these hypotheses appear incongruent with behavioral observations and the animals’ neural constraints. The “Navigation by Scene Familiarity Hypothesis” proposed originally for insect navigation offers an elegantly simple solution for retracing previously experienced routes without the need for complex neural architectures and memory retrieval mechanisms. This hypothesis proposes that an animal can return to a target location by simply moving toward the most familiar scene at any given point. Proof of concept simulations have used computer-generated ant’s-eye views of the world, but here we test the ability of scene familiarity algorithms to navigate training routes across satellite images extracted from *Google Maps*. We find that Google satellite images are so rich in visual information that familiarity algorithms can be used to retrace even tortuous routes with low-resolution sensors. We discuss the implications of these findings not only for animal navigation but also for the potential development of visual augmentation systems and robot guidance algorithms.

## Introduction

Natural selection has sculpted sensory systems and brains such that they can coordinate navigation tasks quickly, repeatedly, and with little error. Of particular interest are small-brained insects, which by virtue of their inherent parsimony may offer insights for understanding the first principles of navigational algorithms [1,2]. Deciphering the algorithms inside a navigating insect’s head might also bring societal benefits such as the development of smart autonomous vehicles and technology to augment the sensory and navigational abilities of visually impaired individuals.

For example, a foraging ant or a worker honeybee precisely navigates long distances from nest to food and back [3,4]. How can circuitry capable of such a complex task be contained in less than a cubic millimeter of brain tissue? Many hypotheses have been proposed to suggest how these insects navigate, ranging from sequential recall and template matching of stored visual images [5] to a human-like map sense [6,7]. The storage of long, accurately ordered sequences of views, or of the complex relationships between them (allocentric representation), however, would seem to require complex circuitry and has proven difficult to implement in closed loop models [8]. What’s more, such schemes seem to rely on anthropocentric ideas of how humans might design a navigation system.

In general, the constraints imposed by the limited neural resources of insects seem to promote the use of efficient task-dependent solutions to sensorimotor challenges [9,10,11]. In this spirit, the Navigation by Scene Familiarity Hypothesis (NSFH) was developed as a model of route navigation in ants [12,13,14]. NSFH is an egocentric algorithm that does not require storage of complex relational memories among visual scenes. The visual information required to navigate a route is stored in a single neural network, which after route learning can output a familiarity score for any view presented to it. Subsequently, the visual information of a perceived scene addresses the memory directly and navigation is achieved implicitly by using a trained network to assess the familiarity of visual scenes, thus enabling a search for familiar scenes. Baddeley et al. [13] produced a virtual agent that used an NSFH-inspired algorithm to retrace visual routes consisting of computer-simulated silhouettes of bushes, trees, and tussocks. The agent generated routes that shared many characteristics with those documented in field studies of navigating ants [15,16,17].

Such parsimonious algorithms can work because of the structured learning made possible by redundancy within navigational systems. In insects and many animals, for instance, path integration [18,17] can guide direct routes through unfamiliar terrain, providing an opportunity to learn visual information [19]. Similarly, insects have dedicated learning behaviors, called learning flights or learning walks, which also provide ideal opportunities to learn visual information [20,21,17]. In these cases, some of the computational load associated with navigational learning is outsourced to innate behaviors that position the agent at the correct place to learn simple views.

The aim of this work was to test the NSFH approach for a novel visual ecology, namely that produced by an aerial platform with a downward facing, low-resolution visual sensor. To provide a simple and flexible simulation we used satellite images taken from Google Maps. Google Maps is a rich, readily accessible source of satellite images that can be scaled to various altitudes and used to test an artificial agent’s tracking proficiency across a range of landscapes and sensor morphologies. While other studies have used satellite images, maps, and panoramic street views for localization and navigation [22,23,24], we use an insect-inspired approach to assess scene familiarity by computing pixel-by-pixel scene differences from a fixed sensor matrix.

A premise of the NSFH is that natural scenes are sufficiently rich with information that even an agent with a low-resolution sensor will not get confused and follow an incorrect path. We developed MATLAB scripts to access Google satellite images, analyze their information content given sensors of various complexities, and test the ability of hypothetical autonomous agents to retrace various training routes. We emphasize that the point of this work is not to optimize the navigational algorithms. Rather our aim is to show that downward facing views of natural scenes contain sufficient information for route navigation via NSFH-style algorithms, and to investigate how navigation performance depends on the interplay between environment and sensor resolution.

## Methods

### Accessing Google Maps

We obtained an application programming interface key by registering with Google Maps at https://code.google.com/apis/console, which then allows 25,000 map requests per day. We wrote a MATLAB script that allows the user to choose the latitude and longitude of desired scenes and the level of zoom of the image, from 0 (whole world) to 21 (very close up), the map type (roadmap, satellite, terrain, hybrid), and the image format (png/png8, png32, gif, jpg, jpg-baseline). In the examples described here, we chose the satellite map type and a zoom level of 18 (~ 250 m above the ground); we saved our images in the png32 format.

We centered our main study landscape on the Meeting House on the University of Sussex campus, UK, which is located at longitude 50.8649 and latitude -0.0880. We stitched together nine contiguous scenes (3 x 3 arrangement), after cropping out the Google watermark from the bottom of each scene. The final product was a 3600 x 3600 pixel satellite image of the Sussex campus and some of the surrounding forest and fields.

In addition to the Sussex landscape, we chose and constructed additional landscapes representing a range of visual complexities. Our simple landscape was a patch of open water in the middle of Lake Derwentwater, south of Keswick, UK (longitude 54.5739, latitude -3.1476); our medium landscape was a sandy region dotted with vegetated hummocks near Kelso Dunes in the Mojave National Preserve, USA (longitude 34.9410, latitude -115.8234); our complex landscape was centered on the Anne Frank House in Amsterdam, NL (long

### Visual System

Our visual system acted as a downward facing “beam” that detected a 640 x 640 pixel scene from the larger 3600 x 3600 satellite image and spanned approximately 100 m of land surface (a 100 m patch of land from 250 m in height represents a beam of 22.6° in width). We applied the MATLAB function “histeq” to enhance contrast using histogram equalization; analogous processes occur in many animals that use temporal and/or spatial neural summation to enhance contrast [25].

**Fig 1:**
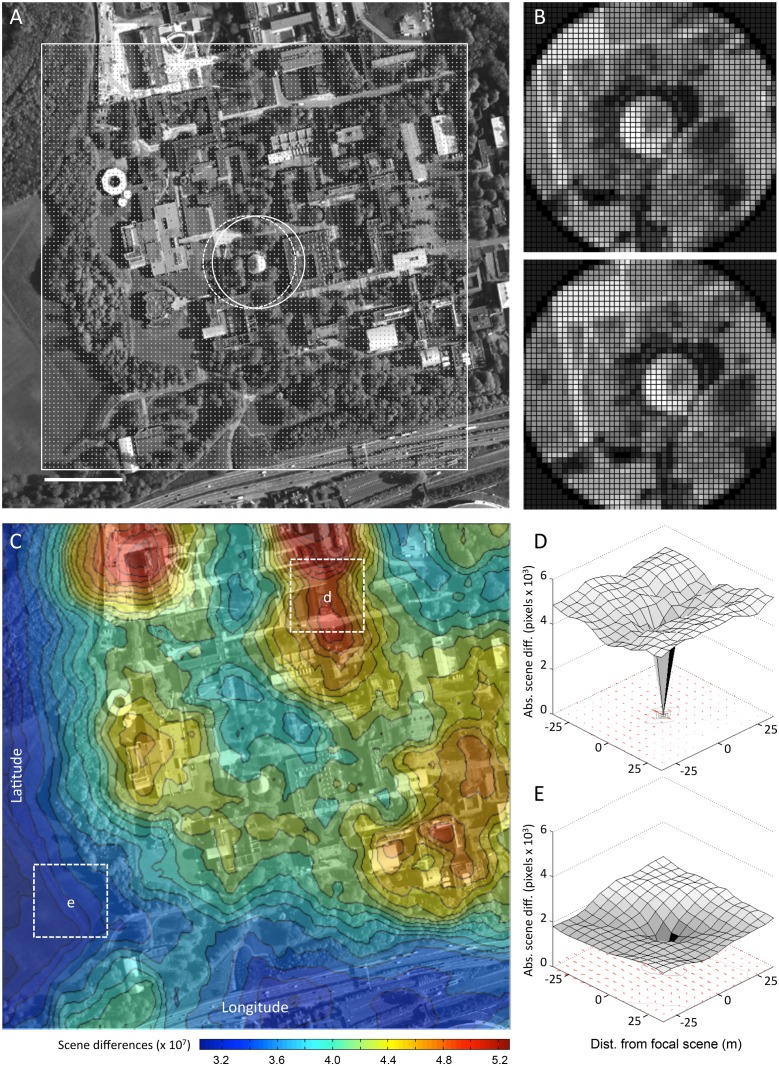
Scene familiarity landscapes from satellite images. **A** Satellite images (zoom level = 18; ~250 m camera altitude) of an area encompassing the University of Sussex campus in Falmer, England were stitched together in MATLAB to form a single 3,600 x 3,600 pixel grayscale image (scale bar = 100 m). The focal scene (highlighted by the right-most central white circle) is centered on the Meeting House on the Sussex campus (longitude: 50.8649; latitude: -0.0880). Ten thousand 640x640 scenes were sampled at equally spaced points in a 100x100 grid (white dots) across the larger landscape. Each scene was processed to 50 x 50 resolution with 10 levels of gray. These sample scenes were circularized prior to similarity processing. **B** Expanded views of the central scene and a scene shifted two sampling points to the left. These locations are indicated by white circles in **A**. **C** All 10,000 scenes in **A** were compared to all other scenes. The contour plot indicates average image difference values by scene position. The plot in **C** is superimposed atop the campus satellite image for reference. Dotted rectangles **d** and **e** show regions of relatively high and relatively low distinctness. These two positions are used to generate the volcano plots shown in **D** and **E**. These plots show the absolute pixel-by-pixel image differences of the focal scene compared to the surrounding 225 scenes (15 x 15 square). The red arrows below the volcanoes indicate the orientation of the best-matched surrounding scene rotated through 360 degrees and compared to the static focal scene; arrow length varies directly with the goodness of the match. Satellite images used under a CC BY license, with permission from Esri, original copyright 2015.

We also pixelated each scene to a specific matrix size and pixel depth (number of gray levels). For example, in the landscape shown in Fig 1A, we reduced the resolution of each scene to a 50 x 50 pixel matrix, with each pixel scaled to 10 levels of gray. However, to compare sensor resolutions we also varied the pixel densities (10 x 10, 20 x 20, 40 x 40, 80 x 80) and pixel depth (black/white, 10 gray levels, 100 gray levels) during scene processing. The corners of these matrices were cropped to form circles so that we could more easily compare directional information between scenes (Fig 1B). In the example of Fig 1A and 1B, circularization reduced pixel coverage by 21.5% (from 2,500 to 1,963 pixels). The result is a low-resolution sensor with limited field of view and no distortion due to perspective.

### Landscape Information Analysis

To assess the familiarity of different parts of the surveyed region, we first extracted 10,000 circularized scenes in a 100 x 100 grid (Fig 1A). The centers of adjacent scenes were displaced by about 5 m, resulting in 95% overlap between neighbors. The similarity or familiarity of each scene is then calculated by comparing it to all other scenes on a pixel-by-pixel basis with the absolute difference between pixels summed to generate a relative scene-to-scene difference score, the *image difference* score, which is summed across all 10,000 scenes to give a 100 x 100 image difference topography map of the surveyed region (Fig 1C). In this map the greater the difference value at a given point, the greater the difference of the 1,963 pixels in the scene at that point to the same pixels of all of the other 9,999 scenes. A high difference value therefore represents a scene that is distinct relative to other scenes in that environment.

The image difference between the scene viewed currently and a stored scene can be used to guide spatial behavior in two ways [26,27]. Because image difference between scenes oriented in the same direction increases with distance between them, a single scene can be an attractor to the location it was stored at. To visualize the use of a stored view in this way, in Fig 1D and 1E we show the image difference between focal and surrounding views which create surface “volcanoes.” If an agent maintains a constant heading and moves to reduce the difference between current and stored scenes, it will navigate down the surface in a form of gradient descent. From a given surface plot we can thus gauge how useful a stored image would be as an attractor by looking for smooth gradients in image differences in the region around the image. Alternatively, a scene can be used as a “visual compass;” that is, the orientation of a stored focal scene can be recovered by rotating the current scene until it best matches the stored scene. We can visualize this information by finding the minima in image differences between a focal scene and systematically rotated scenes (the rotational image difference function, RIDF) from locations surrounding the focal location and plotting the resultant directions as arrows below the surface volcanoes (Fig 1D and 1E).

**Fig 2:**
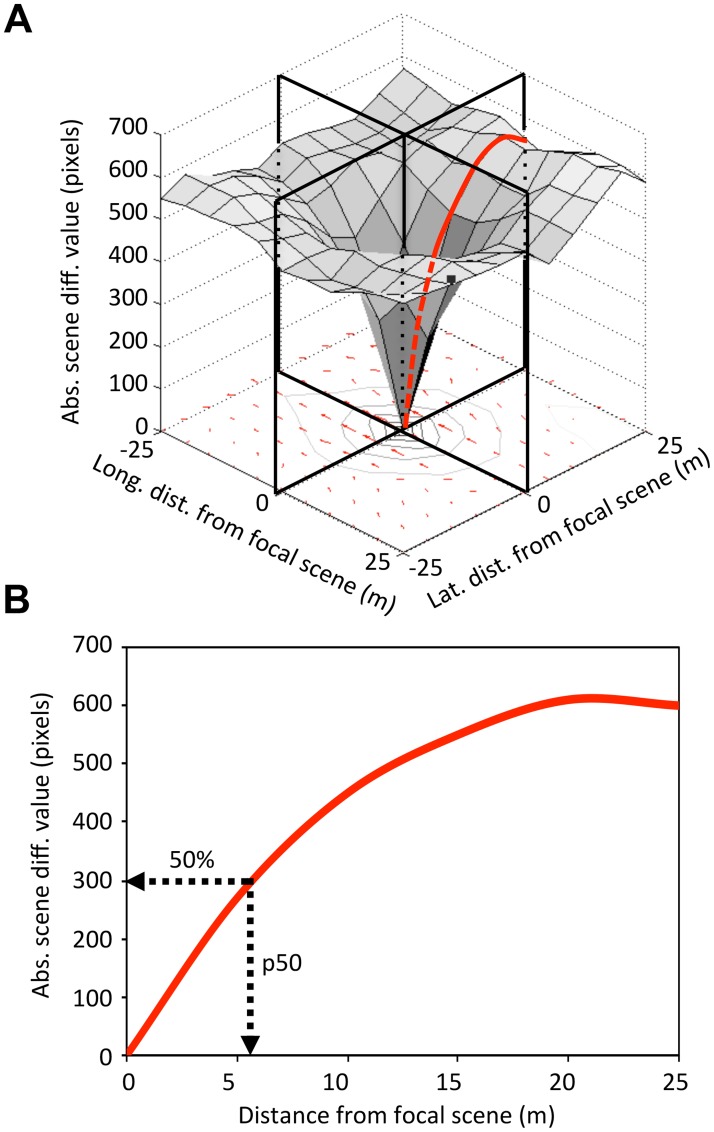
Comparator measures of landscape information. **A** Slices are made through the familiarity volcano in the four cardinal directions and the values are averaged to produce the curve shown in **B**. The absolute scene difference value and the distance from the focal point (in meters) are derived from this curve at the 50% maximal difference point (p50).

To assess scene information among various landscapes, we used volcano plots and RIDF analyses for the Derwentwater, Mojave, and Amsterdam satellite regions. In each case, we used 100 x 100 scene spacing and compared 400 adjacent scenes (in a 20 x 20 square) to five focal scenes. To calculate comparator measures of landscape information, we first made perpendicular slices through the volcanoes (Fig 2A) and averaged the absolute scene difference values by distance from the focal scene for the four directions (Fig 2B). We then determined the absolute scene difference value and the distance from the focal point (in meters) at the 50% maximal difference point (p50) on the resultant curve. To assess visual compass information, we calculated RIDFs for a location by rotating the scene 360 degrees in one-degree increments, comparing each to the focal scene. Volcanoes and RIDFs are averaged from values obtained from five evenly spaced focal scenes from across the region (specifically, by row and column: 25,25; 25,75; 50,50; 75,25; 75,75).

### Route following Algorithm

We developed and tested several algorithms during the course of these studies. We describe here our best prototype based on speed and accuracy of route retracing. We note that the purpose of this paper is not to develop an algorithm with arbitrarily good performance; rather we use the performance of route following algorithms as proof that the information within this visual ecology is sufficient for navigation using a familiarity algorithm.

#### Creating the training path

**Fig 3:**
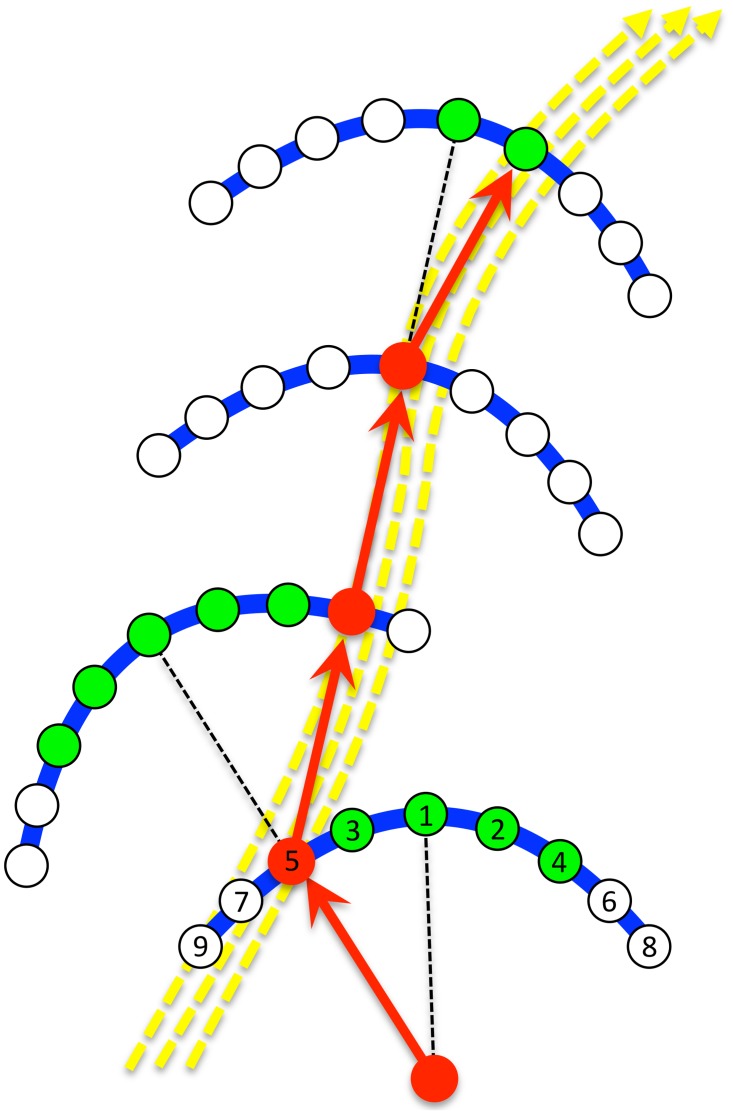
Schematic of route tracking algorithm. The yellow dotted arrows indicate a user-selected route. The blue arc scans are based on the direction of the most familiar scene at that point (dotted black lines). Initial samples are made in the direction of the current path with subsequent samples taken at progressively wider angles on the arc, alternating left and right views (green points). At each point sampled, the agent rotates the focal current scene through 360 degrees and compares each rotation to all stored memory scenes (yellow path). Once a familiarity threshold is met, no further points on the arc are sampled. The agent moves forward to this best-matched point (red line) and casts its next scan based on an extension of this red line segment (see Methods for further description).

The program presents the selected satellite region and queries the user to select the number of points to be marked in the training path. Cross hairs appear over the satellite image to guide the user’s placement of training points. Once all of the training points are placed, the program interpolates and plots the contiguous points between each successive point to create a continuous line for the main path and then increases the width of this path by a scene on either side (yellow dashes, Fig 3). The bearing of travel between successive user-placed points is then determined based on a 360-degree compass system with north being zero. To simulate what the agent would “see” as it made its way along the path, each scene in the training path is then rotated by this bearing and stored in memory.

#### Recapitulating the training path

To initiate recapitulation, the user places the agent near the beginning of the learned route, which is akin to an ant or a bee either leaving its hive for a journey to a learned food source, or leaving a food source, to return home. Ants are known to scan the world when in an unfamiliar location [28] and this behavior inspires our agent’s strategy. The agent’s first scan is based on the direction of the most familiar scene at that point. The agent produces a scan in an arc (blue lines, Fig 3) whose size varies depending on a user-set value (here we used a value that produced an arc that spanned about 40 m of ground surface). The initial point sampled on the arc is in the direction of the current path with subsequent samples taken at progressively wider angles from this direction, alternating the left and the right sides (green points, Fig 3).

At each point sampled, the agent rotates its current focal scene through 360 degrees at user-defined increments, comparing each incremental rotation to all stored memory scenes. If a user-set threshold of familiarity is met (here we used a threshold of 20% of the mean of difference values obtained by comparing all rotations of the current surveyed point to all scenes in the training path), then no further points on the current arc are sampled and the agent moves forward to this best-matched point (red line, Fig 3). It casts its next scan based on the forward extension of the red line segment (dotted black lines, Fig 3). The program continues until the re-tracked path is within a designated distance of the end of the training path, runs off the satellite area, or is terminated by the user.

## Results

### Scene Information Analysis

**Fig 4:**
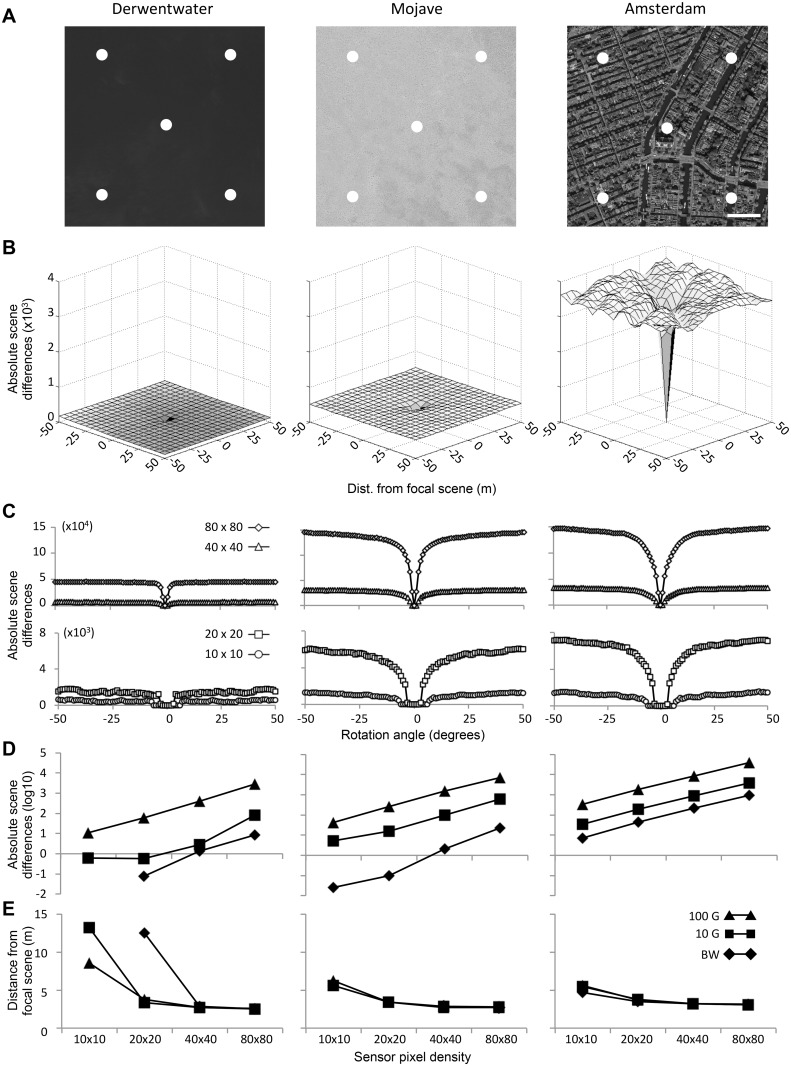
The spatial information available in natural scenes. **A** Five sampled focal scenes are shown as white dots superimposed on satellite regions for Derwentwater, Mojave, and Amsterdam (scale bar = 100 m). **B** The averaged volcano plots for these five scenes, each comparing focal scenes to the surrounding 400 scenes (20 x 20 grid) are shown for a 20 x 20 pixel-resolution with 100 gray level pixel depth. A common y-axis is used to show information differences among regions. **C** RIDF curves averaged across the five landscape sample points for the four sensor resolutions indicated in the legend (all at 100 gray level pixel depth). Absolute scene difference information (**D**) and distance from focal scene (**E**) are plotted for each region for each of the sensor resolutions and pixel depths as calculated at the p50 position on the information volcanoes (as described in Fig 2). Satellite images used under a CC BY license, with permission from Esri, original copyright 2015.

We explored the potential quality of various scenes for visual navigation by examining the scene difference information as shown by volcano and RIDF plots of the region. Fig 4 shows these analyses for three selected satellite regions of varying complexity. Fig 4B and 4C show sample volcano and RIDF plots averaged from the five points shown in Fig 4A. This simple analysis gives us some sense of how useful a stored view would be for navigation. Volcano plots (Fig 4B) show a central low point whose depth relates to how distinct a view is within that environment. As we would expect the more visually rich scene of Amsterdam provides scenes that are more distinct than the surroundings. The volcano plots show that even the homogenous landscapes of deserts and lakes do provide distinct views, however the minima are shallow.

RIDF analysis (Fig 4C) indicates whether usable directional information remains in the views of a world as produced by a particular sensor. We see that our low-resolution, small beam visual sensor provides navigationally relevant information from our world; information is available even within the relatively featureless Derwentwater landscape using the 80 x 80 sensor (though it should be noted that in a live situation, changing wave patterns, light glint, and shadows should negate visual information present in static satellite images).

Volcano plots also highlight a catchment area whereby one could descend a gradient of the image difference function to return to the location of the stored scene. An analysis of the p50 point of these curves (Fig 4D and 4E) for various sensor designs highlights two key points: (1) lower resolution sensors produce a larger catchment area. And, (2) the number of gray levels does not impact on the catchment area in complex scenes.

Taken together, these scene analyses show that directional and positional information can be derived from views with a range of resolutions and in natural environments. However, high-resolution views show better performance when used as a “visual compass” and as expected, the visually rich environment of Amsterdam also allows for better performance.

### Tests of route recapitulation algorithm

**Fig 5:**
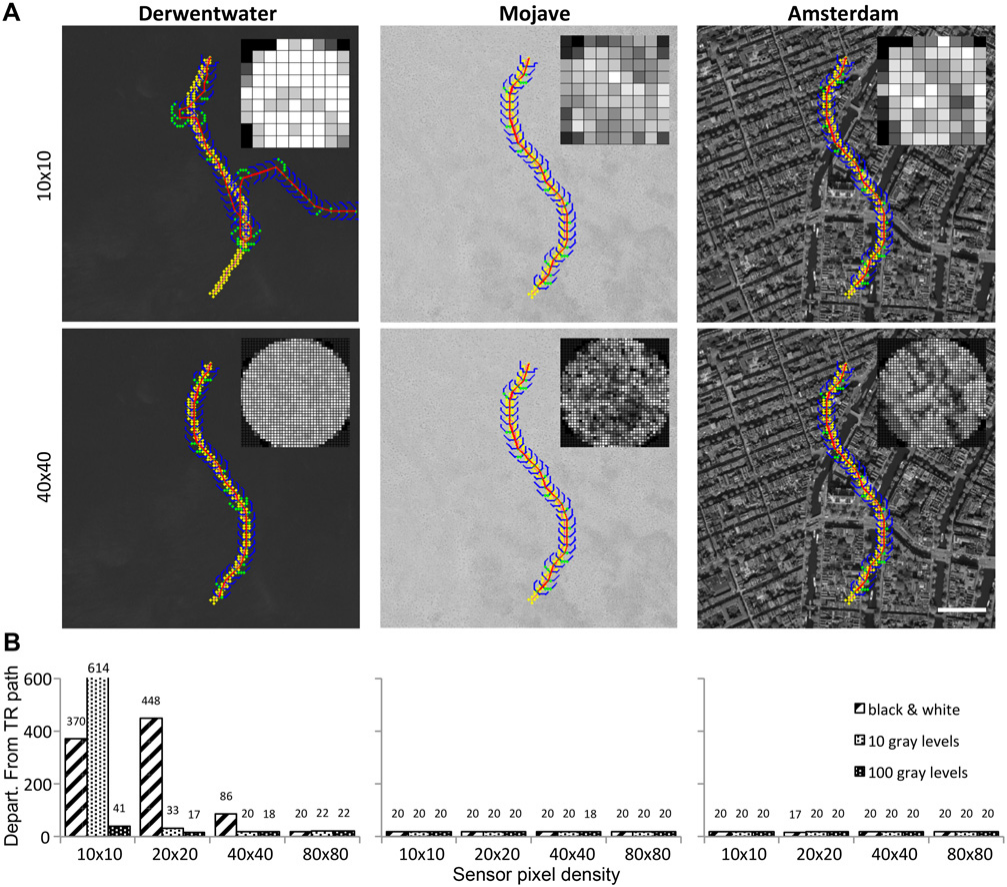
Route recapitulation performance across different landscapes. We tested the ability of the auto-tracking algorithm to retrace simple, S-shaped training routes (yellow dots) drawn across the Derwentwater, Mojave, and Amsterdam satellite regions (scale bar = 100 m). **A** Sample retraced paths are shown for two sensor resolutions (10 x 10 and 40 x 40) and 10 gray level pixel depth (insets show examples of sensor images). **B** Cumulative distance of departure of the retraced route from the training route is shown for the three regions at the four sensor resolutions and three pixel depths. Plotted are the summed geometric distances of each best-matched point (red) to the appropriate training path point (in sequence) based on a 100 x 100 point sampling grid imposed on each region (actual values given above bars). Satellite images used under a CC BY license, with permission from Esri, original copyright 2015.

Having shown that the information for navigation exists, we now turn our attention to whether sensors of this type could be used to form a simple visual route navigation algorithm. However, we reiterate that our goal for this analysis is to show that this information can be used for navigation and not to develop an optimal navigational algorithm. We first tested the performance of the tracking algorithm to navigate a simple S-shaped training path across the three experimental satellite regions. Fig 5A shows sample paths and re-tracings for two sensor resolutions (10 x 10 and 40 x 40; 10 levels of gray). The insets show examples of perceived scenes. Fig 5B summarizes the performance of all four resolutions and three pixel depths across the three regions. The only re-tracings that failed to reach the target were for the 10 x 10 (BW, 10 gray), 20 x 20 (BW), and 40 x 40 (BW) sensors in the Derwentwater region. The 10 x 10 (100 gray) and 20 x 20 (10 gray) sensors meandered a bit, but still reached the target. The Derwentwater 20 x 20 (100 gray), 40 x 40 (10 & 100 gray), and 80 x 80 (BW, 10, & 100 gray) sensors had no difficulty traversing the path correctly. All of the sensors tracked the training path accurately for the Mojave and Amsterdam regions. The histeq transformation improved performance of the auto-tracking algorithm. For example, in these trials the pre-transformation algorithm recapitulated the path correctly in 4 of the 12 pixel density/depth combinations for Derwentwater and 8 of the 12 for Mojave. After transformation, the success rate improved to 8 of 12 (Derwentwater) and 12 of 12 (Mojave).

**Fig 6:**
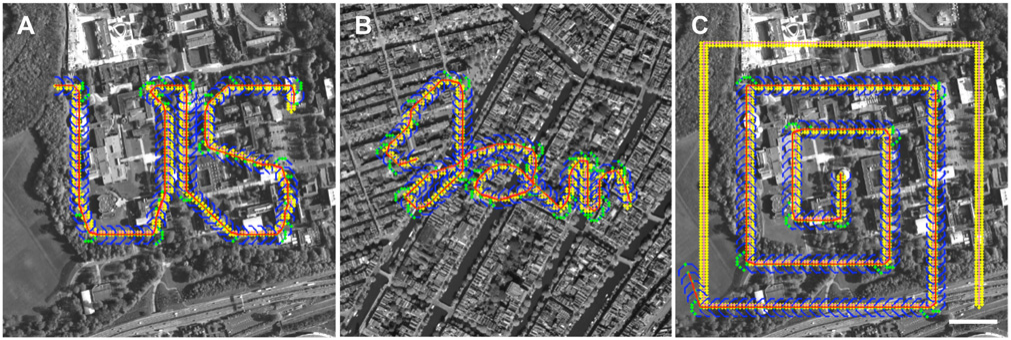
Examples of tracking algorithm performance on complex routes. **A** Retracing of a route in the shape of the University of Sussex logo over the Sussex landscape. This route had several abrupt turns, including one of about 135 degrees. **B** Test of the tracking algorithm on a route that loops across itself. This route spells out a scripted “Jan” in honor of an uncle who lived in this Amsterdam neighborhood. **C** An expanding square spiral beginning at the Meeting House on the Sussex campus. The tracker accurately retraced the route until it passed through the region of low scene difference information over the field at the lower left of the image (near region **e** in Fig 1C; scale bar = 100 m). Satellite images used under a CC BY license, with permission from Esri, original copyright 2015.

We ran several tests of the route algorithm on more complex paths; three of these are shown in Fig 6. We traced the “US” logo for the University of Sussex atop the Sussex campus and retraced the route successfully with a 50 x 50, 100-gray sensor (Fig 6A). The tracker required several samples to negotiate the corners of the pattern and even retraced the 135° turn on the upper right side of the “U.” We also tested the tracker on training routes that contained loops (Fig 6B). Although, such training routes are unlikely in actual homing animals, the tracker is able to retrace these routes because of the directional scene information as computed by the RIDF and the implicit forward momentum of the algorithm. In this situation, our algorithm outperforms that described in Baddeley et al. [13], in which each step is predicated on the compass bearing of the best RIDF match. Here we used a system that sets the next saccade arc based on the line determined by the best matched point on the current saccade, with the pivot determined by the position of the previous best matched point. As such, we take advantage of an aerial agent casting its gaze forward of its current path. This approach makes the agent more likely to follow its current path than that of a crossing path. We also tested the tracker on an expanding square spiral centered over the heterogeneous Sussex campus landscape. The tracker successfully negotiated the right-angle corners in the high information areas above the buildings, but failed in the low information area over the field at the lower left (Fig 6C). This behavior is consistent with the information difference topography shown in Fig 1. Subsequent tests with a more stringent matching threshold allowed the tracker to retrace the complete spiral.

## Discussion

The results of this study are both clear and surprising. We used the Navigation by Scene Familiarity Hypothesis to develop algorithms for the recapitulation of visually guided routes across *Google Maps* satellite images. We show proof of concept that even a simple, low-resolution sensor can navigate complex paths accurately and efficiently. Of course, it must be noted that our analyses depended on restricted visual imagery taken from a perspective higher than where most animals fly. Still, by analyzing the visual information available in satellite images from various regions with different sensor structures we show that the inherent richness of visual scenes suggests that this simple navigation algorithm may be applicable to natural environments as well. What’s more from a biological point of view, this work reinforces a way of thinking about visually guided behavior where tasks such as navigation can be accomplished with coarse visual information and an absence of perceptual processes such as object or place recognition.

### The memory potential of neural tissue

The NSFH model is elegantly simple: The animal or the agent simply flies or moves toward areas of highest familiarity. Of course, this depends on an agent being able to store all the scenes that are associated with a desirable route. Is this possible? Although a bee’s brain is less than a cubic millimeter in volume, the tissue between the eyes and motor centers is complex [10] and has approximately one million neurons [29] and a billion synapses. Therefore even a small brain has the potential to produce a large number of unique codes. Can this account for *all* the ‘glimpses’ that a bee might experience during her one- to two-month foraging lifetime? Active worker honeybees live about six to seven weeks. Rounding up to two months (60 days), multiplying by 24 hours in a day, 60 minutes in an hour, 60 seconds in a minute, and an eye frame capture rate of 60 per second, we get 180x24x60x60x60 = 311,040,000 scenes she could experience in a lifetime. How do these numbers compare to the architecture of a bee’s visual system? Each eye of a honeybee contains about 4500 ommatidia [10]. Visual information from ommatidial receptors relays through massively parallel neuropil structures with synaptic connections in the lamina, medulla, lobula, and among local interneurons before it reaches the mushroom bodies, central complex, and descending neurons.

This visual network thus provides an enormous number of nodes and connections. Take for example, a simple array of two elements with two possible states: “on” or “off.” The number of unique configurations for this array is four ([0 0], [0 1], [1 0], [1 1]) and in general the number of permutations is 2^n^ where n is the number of array elements. Obviously, with a visual system of thousands of ommatidia and tens of thousands of synaptic connections, we have a huge storage potential that exceeds the volume of potential lifetime visual memory required by a foraging insect. This is before one considers the efficiencies in visual coding (as opposed to storing raw pixel images [30]) and in deriving familiarity measures from, for instance, a neural network trained on the perceived images as opposed to storing all of them as a “perfect memory” (see [13]).

These calculations assume that landscapes possess enough visual variability to address this neurological potential. Our scene analyses showed that the visual environment is indeed rich and that even a very simple sensor can access this information and suffer few missteps due to scene aliasing. Indeed even very low-resolution sensors with limited pixel depth produce steep difference information volcanoes when compared to surrounding scenes.

### Potential Applications

We think the NSFH has many potential applications. It is a deliberately simple algorithm and thus is suited to applications where low power and weight are priorities such as autonomous robotics and wearable sensors. For example, this technology could be used to develop guidance aids for the visually impaired in the acquisition, processing, and retracing of real-world visually defined routes. We also see potential for unmanned vehicles (aerial, ground-based, or submarine) to use similar algorithms where map and GPS information is of little use or unreliable (such as space exploration or inside buildings) or in cases where previously captured images are of little or no value due to significant environmental disruptions.

### Increasing Algorithmic Efficiency

The aim of this work was not to develop the most efficient navigational algorithm possible and therefore the current version could be improved in a number of ways. First, increased performance could be achieved by greater leveraging of a flying agent’s perspective, since being able to see the skyline at the horizon would provide strong directional information [27]. Second, an agent could use different resolution strategies depending on its situation. A high-resolution sensor works best when the agent is on the right path, as it decreases the chance of being fooled by a stray aliased scene. On the other hand, when the agent is off course, a low-resolution, wider-field sensor increases the catchment area and improves the chances of getting back on track [31]. Finally, biasing scene selections toward the direction of movement and by restricting the rotational field of view could improve efficiency by requiring fewer scene comparisons and reduce the chance of choosing an incorrect path.

### Summary

In this paper, we have given proof of concept that a deliberately simple visual sensor (a narrow low-resolution beam) can navigate using a familiarity principle in a visual environment (the world of satellite images taken from Google maps) that is quite different from that previously tested (a panoramic ground-level view of a simulated desert). In so doing, we have both shown the generality of the NSFH approach and also demonstrated the rich information available even in low resolution visual scenes that could be exploited by both small-brained animals and autonomous agents.
